# Supplementary material for: The transcriptional corepressor CtBP2 serves as a metabolite sensor orchestrating hepatic glucose and lipid homeostasis
Source: Nat Commun. 2021 Nov 2;12:6315. doi: 10.1038/s41467-021-26638-5 (PMC8563733; doi:10.1038/s41467-021-26638-5)
Supplement: Supplementary file 1 — Supplementary Information [file 41467_2021_26638_MOESM1_ESM.pdf]

Supplementary Information

The transcriptional corepressor CtBP2 serves as a metabolite sensor orchestrating hepatic glucose and lipid homeostasis

Motohiro Sekiya, Kenta Kainoh, Takehito Sugasawa, Ryunosuke Yoshino, Takatsugu Hirokawa, Hiroaki Tokiwa, Shogo Nakano, Satoru Nagatoishi, Kouhei Tsumoto, Yoshinori Takeuchi, Takafumi Miyamoto, Takashi Matsuzaka, Hitoshi Shimano

Additional information

Supplementary Fig. 1-9

Supplementary Data 1, 2

Supplementary Table 1

Supplementary Movie 1,2

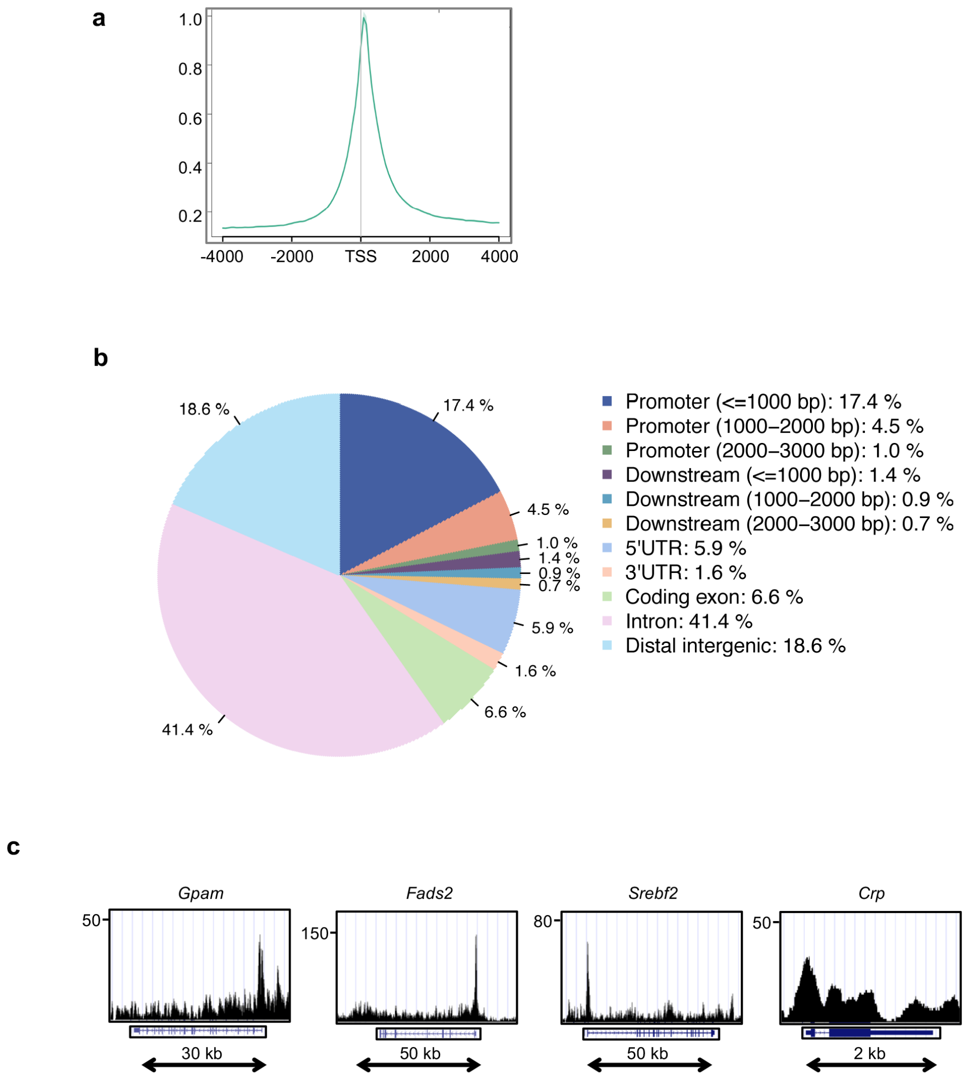
a
1.0
0.8
0.6
0.4
0.2
-4000
-2000
TSS
2000
4000
b
18.6 %
17.4 %
4.5 %
1.0 %
1.4 %
0.9 %
0.7 %
5.9 %
1.6 %
6.6 %
41.4 %
Promoter (<=1000 bp): 17.4 %
Promoter (1000–2000 bp): 4.5 %
Promoter (2000–3000 bp): 1.0 %
Downstream (<=1000 bp): 1.4 %
Downstream (1000–2000 bp): 0.9 %
Downstream (2000–3000 bp): 0.7 %
5'UTR: 5.9 %
3'UTR: 1.6 %
Coding exon: 6.6 %
Intron: 41.4 %
Distal intergenic: 18.6 %
c
Gpam
Fads2
Srebf2
Crp
50
150
80
50
30 kb
50 kb
50 kb
2 kb

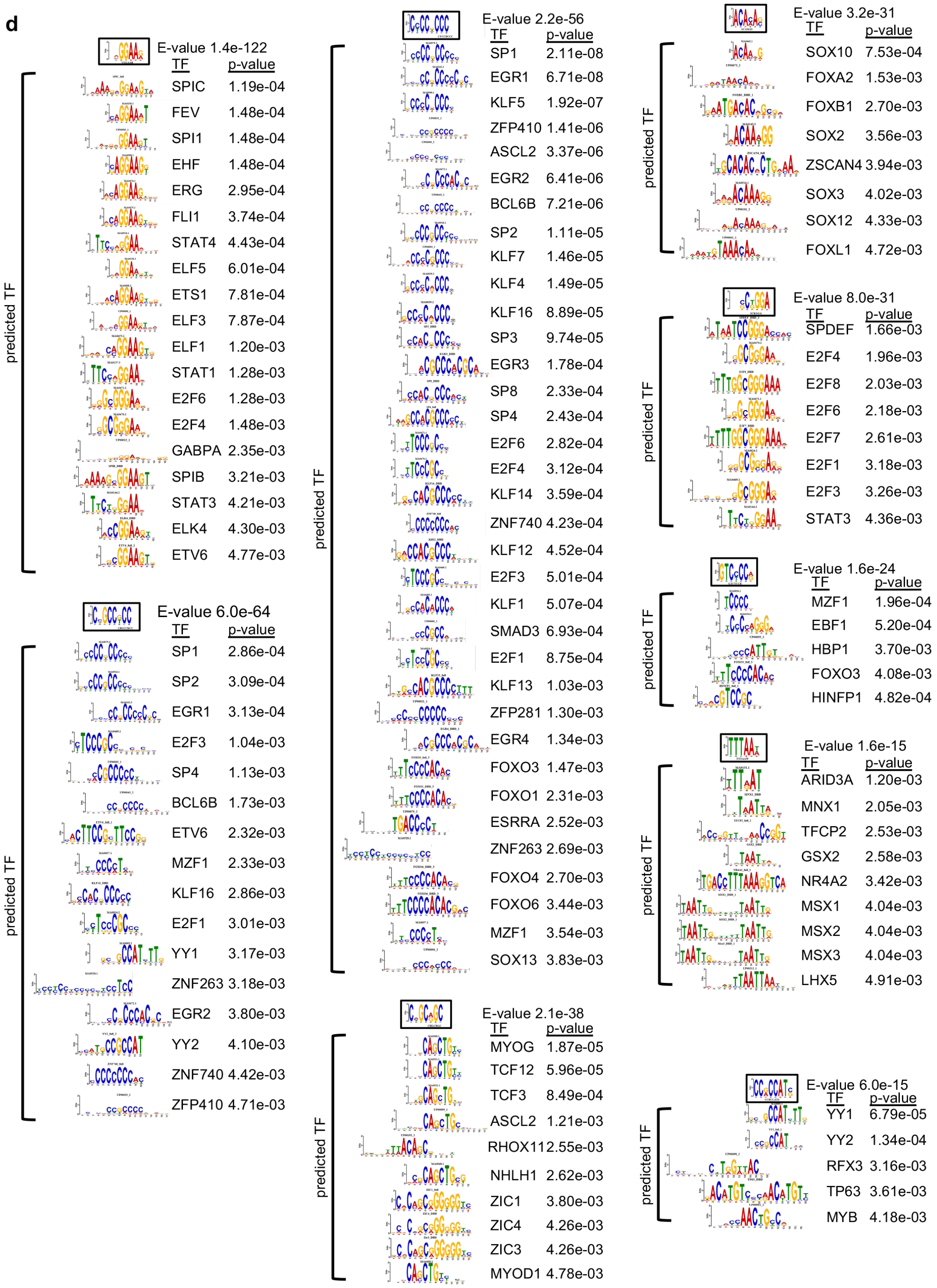
d
E-value 1.4e-122
TF p-value
SPIC 1.19e-04
FEV 1.48e-04
SPI1 1.48e-04
EHF 1.48e-04
ERG 2.95e-04
FLI1 3.74e-04
STAT4 4.43e-04
ELF5 6.01e-04
ETS1 7.81e-04
ELF3 7.87e-04
ELF1 1.20e-03
STAT1 1.28e-03
E2F6 1.28e-03
E2F4 1.48e-03
GABPA 2.35e-03
SPIB 3.21e-03
STAT3 4.21e-03
ELK4 4.30e-03
ETV6 4.77e-03
predicted TF
E-value 6.0e-64
TF p-value
SP1 2.86e-04
SP2 3.09e-04
EGR1 3.13e-04
E2F3 1.04e-03
SP4 1.13e-03
BCL6B 1.73e-03
ETV6 2.32e-03
MZF1 2.33e-03
KLF16 2.86e-03
E2F1 3.01e-03
YY1 3.17e-03
ZNF263 3.18e-03
EGR2 3.80e-03
YY2 4.10e-03
ZNF740 4.42e-03
ZFP410 4.71e-03
predicted TF
E-value 2.2e-56
TF p-value
SP1 2.11e-08
EGR1 6.71e-08
KLF5 1.92e-07
ZFP410 1.41e-06
ASCL2 3.37e-06
EGR2 6.41e-06
BCL6B 7.21e-06
SP2 1.11e-05
KLF7 1.46e-05
KLF4 1.49e-05
KLF16 8.89e-05
SP3 9.74e-05
EGR3 1.78e-04
SP8 2.33e-04
SP4 2.43e-04
E2F6 2.82e-04
E2F4 3.12e-04
KLF14 3.59e-04
ZNF740 4.23e-04
KLF12 4.52e-04
E2F3 5.01e-04
KLF1 5.07e-04
SMAD3 6.93e-04
E2F1 8.75e-04
KLF13 1.03e-03
ZFP281 1.30e-03
EGR4 1.34e-03
FOXO3 1.47e-03
FOXO1 2.31e-03
ESRRA 2.52e-03
ZNF263 2.69e-03
FOXO4 2.70e-03
FOXO6 3.44e-03
MZF1 3.54e-03
SOX13 3.83e-03
predicted TF
E-value 2.1e-38
TF p-value
MYOG 1.87e-05
TCF12 5.96e-05
TCF3 8.49e-04
ASCL2 1.21e-03
RHOX11 2.55e-03
NHLH1 2.62e-03
ZIC1 3.80e-03
ZIC4 4.26e-03
ZIC3 4.26e-03
MYOD1 4.78e-03
predicted TF
E-value 3.2e-31
TF p-value
SOX10 7.53e-04
FOXA2 1.53e-03
FOXB1 2.70e-03
SOX2 3.56e-03
ZSCAN4 3.94e-03
SOX3 4.02e-03
SOX12 4.33e-03
FOXL1 4.72e-03
predicted TF
E-value 8.0e-31
TF p-value
SPDEF 1.66e-03
E2F4 1.96e-03
E2F8 2.03e-03
E2F6 2.18e-03
E2F7 2.61e-03
E2F1 3.18e-03
E2F3 3.26e-03
STAT3 4.36e-03
predicted TF
E-value 1.6e-24
TF p-value
MZF1 1.96e-04
EBF1 5.20e-04
HBP1 3.70e-03
FOXO3 4.08e-03
HINFP1 4.82e-04
predicted TF
E-value 1.6e-15
TF p-value
ARID3A 1.20e-03
MNX1 2.05e-03
TFCP2 2.53e-03
GSX2 2.58e-03
NR4A2 3.42e-03
MSX1 4.04e-03
MSX2 4.04e-03
MSX3 4.04e-03
LHX5 4.91e-03
predicted TF
E-value 6.0e-15
TF p-value
YY1 6.79e-05
YY2 1.34e-04
RFX3 3.16e-03
TP63 3.61e-03
MYB 4.18e-03
predicted TF

1

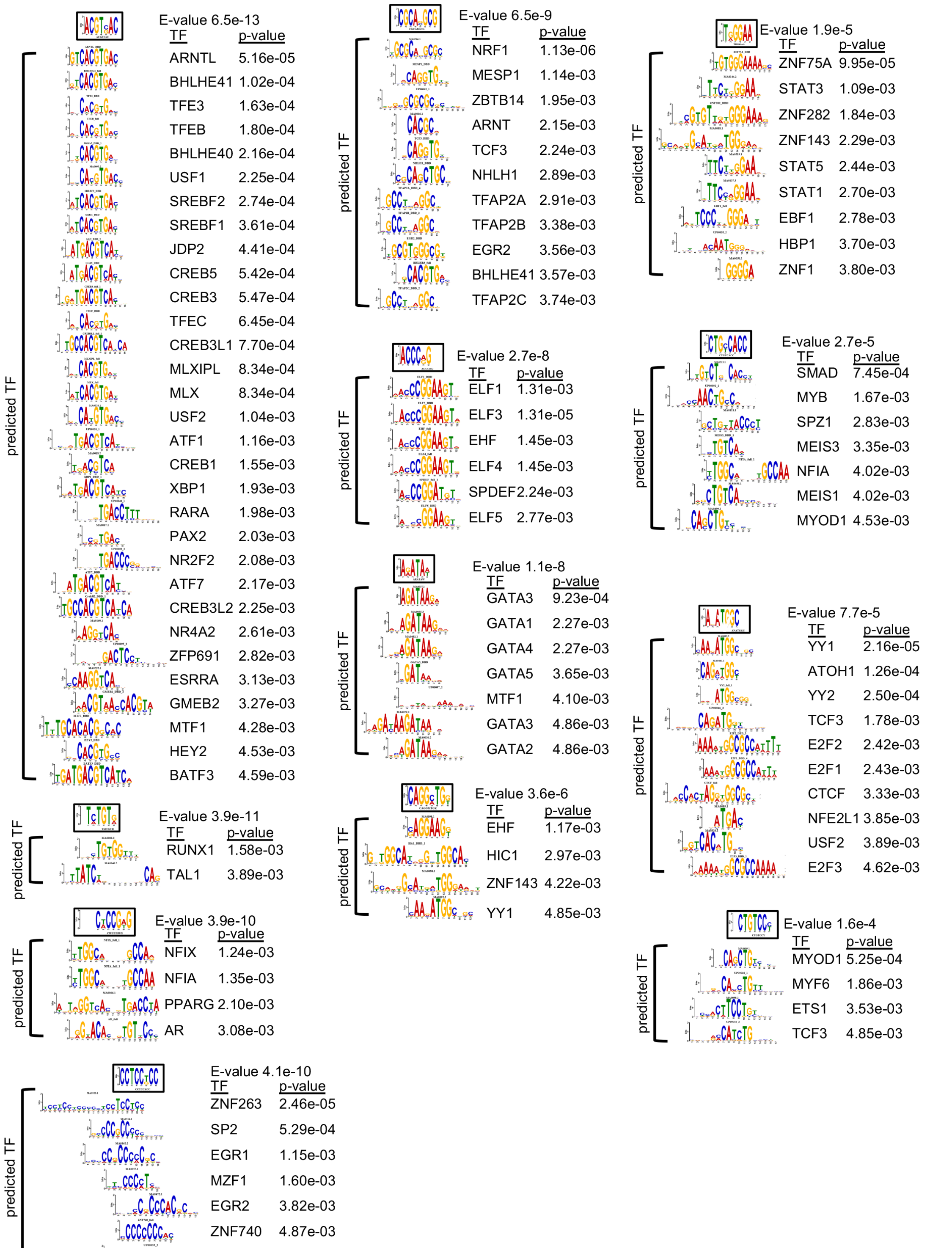

Supplementary Figure 1

Supplementary Fig. 1. CtBP2 cistrome analyzed by ChIP-seq.

a,**b.** Distribution of CtBP2 ChIP-seq peaks in normal liver tissues (6-h fasted) relative to the TSSs (**a**) and percentage distribution of ChIP-seq peaks with

respect to gene features (**b**). **c.** CtBP2 ChIP-seq peaks at representative metabolic gene loci. **d**. Motif analysis of CtBP2 ChIP-seq. The motifs enclosed by a rectangle are CtBP2-binding motifs enriched in the ChIP-seq. Prediction of known transcription factors targeted by CtBP2 based on sequence similarity are shown below [85].

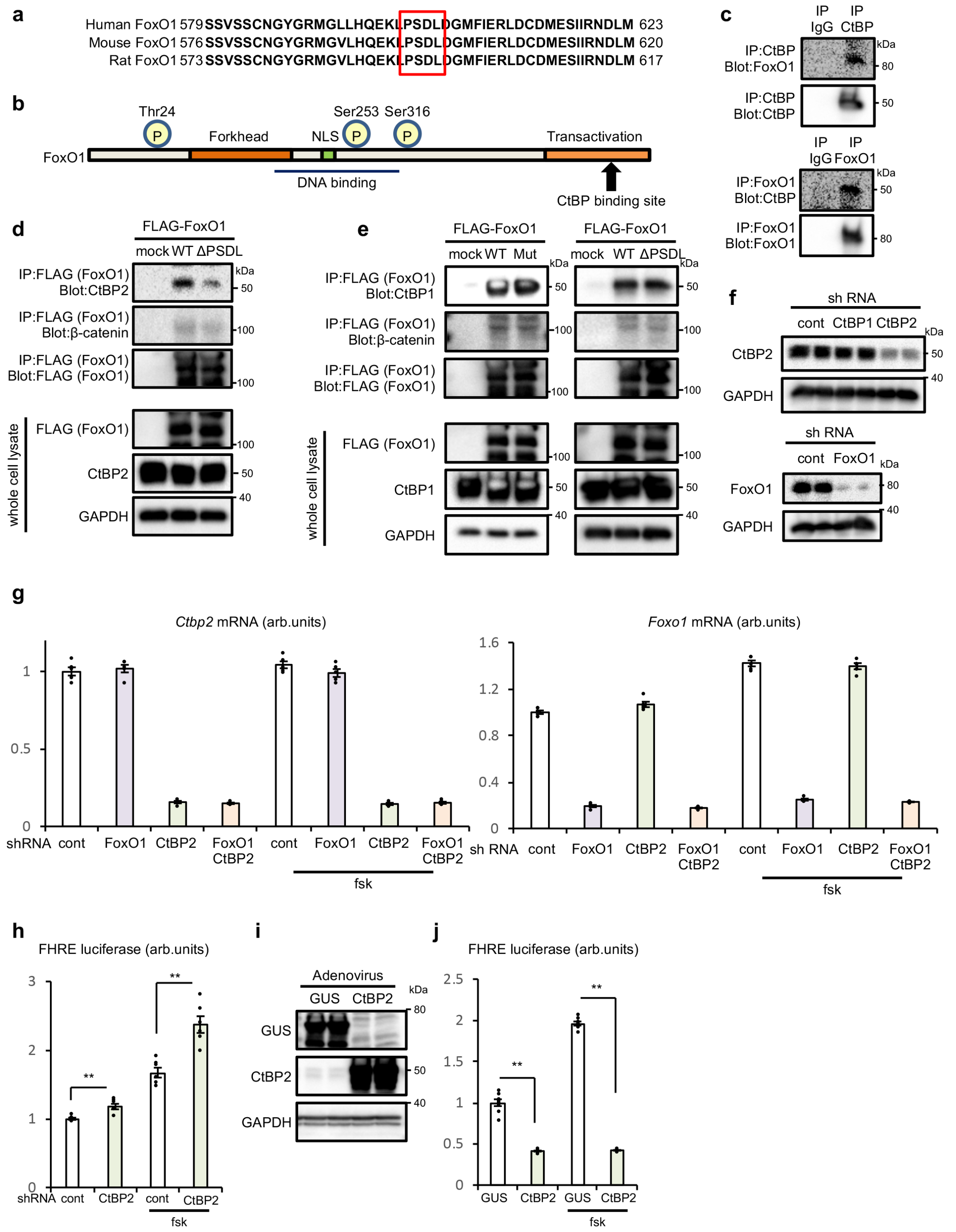

Supplementary Figure 2

Supplementary Fig. 2. Identification of CtBP2/FoxO1 complex as a regulatory system for hepatic gluconeogenesis.

a. The conservation of a PSDL motif in FoxO1 sequences. The red rectangle indicates the location of the motif. **b.** Schematic description of CtBP-binding site in FoxO1. Three representative phosphorylation sites are shown (P). NLS: nuclear localization signal. **c.** Endogenous CtBPs/FoxO1 complex in primary hepatocytes. The CtBP antibody recognizes both CtBP1 and CtBP2 isoforms. **d**,**e.** HEK293 cells were transfected with either control plasmid, FLAG wild-type FoxO1 or mutant FLAG-FoxO1 (Mut: PSDL>PSAS, ΔPSDL: PSDL motif deletion) along with CtBP1 or CtBP2 expression plasmid. The complexes were immunoprecipitated with FLAG magnetic beads. **d.** Deletion of the PSDL motif in FoxO1 diminishes CtBP2/FoxO1 interaction in HEK293 cells. **e.** Mutation or deletion of PSDL motif does not alter CtBP1/FoxO1 interaction. **f,g.** Knockdown efficiencies of shCtBP2 and shFoxO1 adenoviruses at protein (**f**) and mRNA (**g**, same experimental settings as described in Fig. 1e, n=5, biologically independent cells) levels. fsk: forskolin. **h.** FHRE luciferase activity following *CtBP2* knockdown (n=8, biologically independent cells, p-values are 1.9×10^{-3} and 5.8×10^{-4} for sh-cont vs sh-*CtBP2* and sh-cont/fsk vs sh-*CtBP2*/fsk, respectively). **i.** Exogenous expression of GUS and CtBP2 at protein levels. **j.** FHRE luciferase activity (n=8, biologically independent cells, p-values are 1.2×10^{-9} and 2.1×10^{-18} for GUS vs CtBP2 and GUS/fsk vs CtBP2/fsk, respectively) following CtBP2 overexpression. Data are expressed as the mean ± SEM. ** denotes $p < 0.01$ evaluated by unpaired two-tailed Student's t-test. Source Data are provided as a Source Data file.

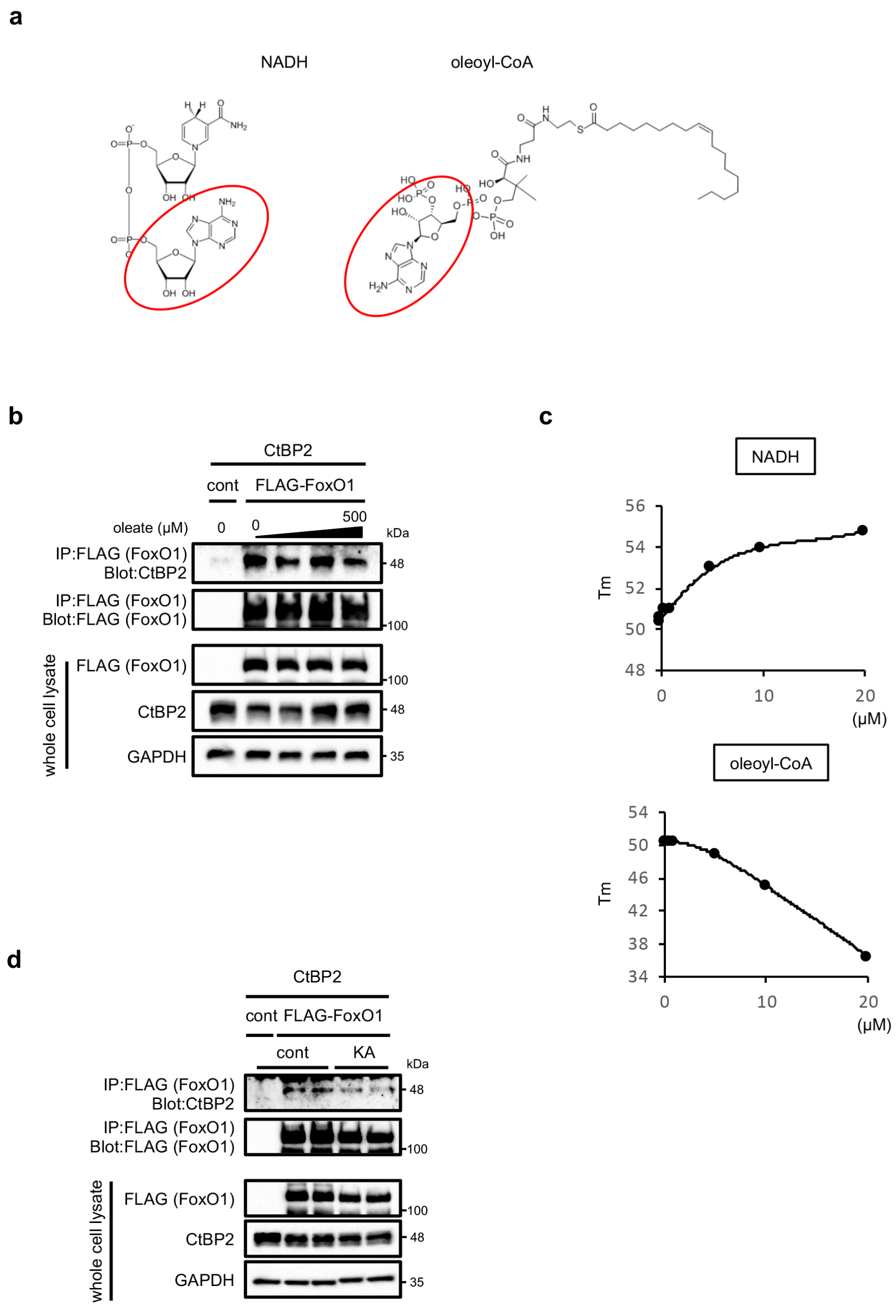

Supplementary Figure 3

Supplementary Fig. 3. Metabolite-sensing capabilities of CtBP2.

a. Structures of NADH and oleoyl-CoA. Adenosine structures are enclosed by red ovals. **b.** CtBP2 does not respond to fatty acids. Increasing concentrations

of oleate (0, 50, 150, 500 µM) were added to the HEK293 lysates. **c**. The metabolite concentration-dependent shift of melting temperatures (Tm) in the DSF assay for a rough estimation of Kd. **d.** The effect of reduced NADH production by chemical inhibition of GAPDH. HEK293 cells transfected with FLAG-FoxO1 and CtBP2 were treated with 5 µM koningic acid (KA, a GAPDH-specific inhibitor) for 5 h. Source Data are provided as a Source Data file.

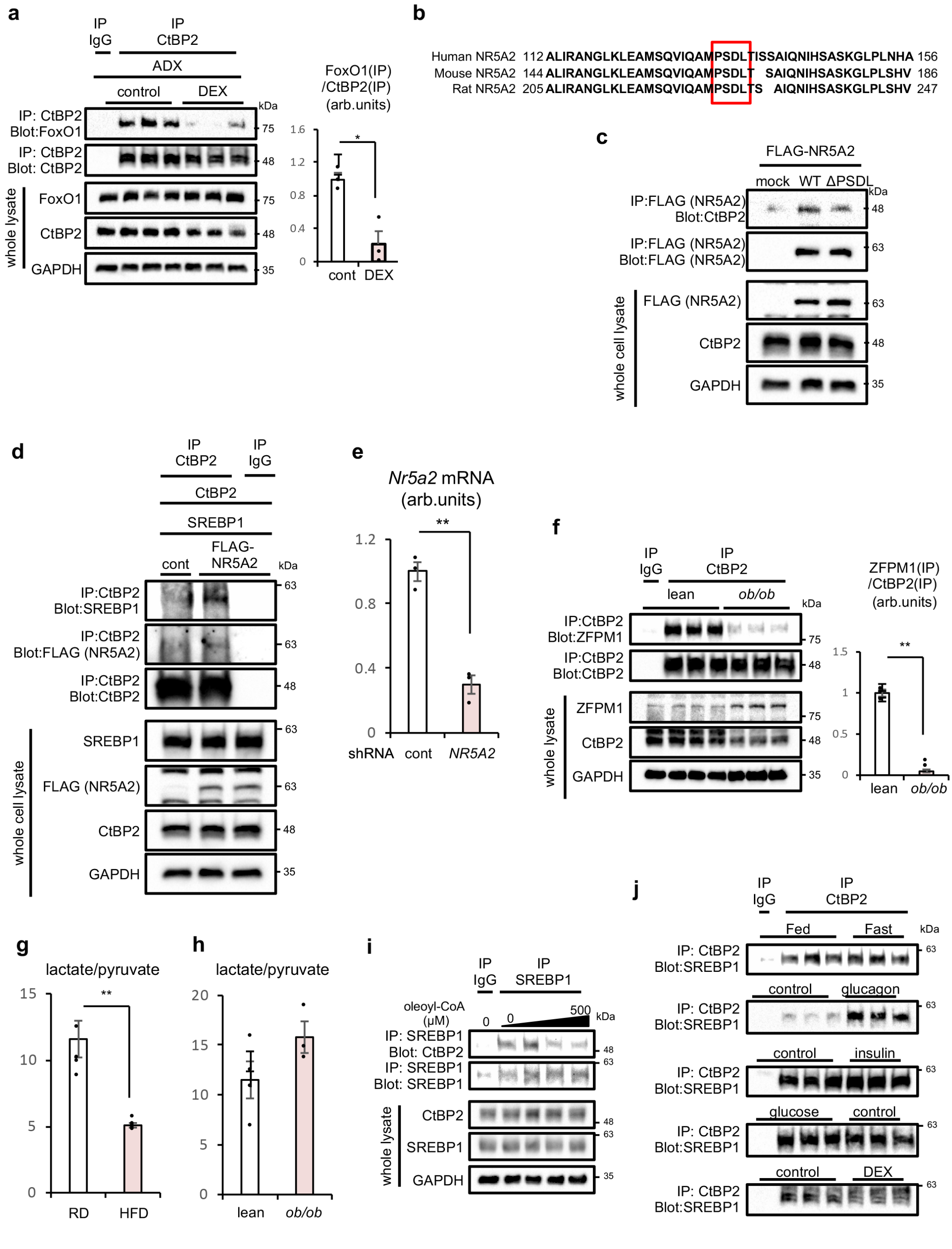

Supplementary Figure 4

Supplementary Fig. 4. CtBP2 complex formation in vitro and in vivo.

a. CtBP2/FoxO1 complex in response to glucocorticoid administration in adrenalectomized (ADX) mice (6 h following injection of 1 mg/kg of dexamethasone, DEX, fed ad libitum, n=3 or 4 biologically independent animals for each group as shown in the blot, $p = 0.011$). **b.** The conservation of a PSDL motif in NR5A2(LRH-1) sequences. The red rectangle indicates the location of the motif. **c.** HEK293 cells were transfected with either control plasmid, FLAG wild-type NR5A2(LRH-1) or mutant FLAG-NR5A2(LRH-1) (ΔPSDL: PSDL motif deletion) along with CtBP2 expression plasmid. The complexes were immunoprecipitated with FLAG magnetic beads. **d.** HEK293 cells were transfected with either control plasmid, FLAG wild-type NR5A2(LRH-1), SREBP1 along with CtBP2 expression plasmid as indicated and immunoprecipitated with either control IgG or anti-CtBP2 antibody. **e.** mRNA expression levels of *Nr5a2* in Fig. 3l (n=3 biologically independent animals for each group, $p = 1.0 \times 10^{-3}$). **f.** Liver homogenates from genetically obese mice (6-h fasted) were subjected to co-immunoprecipitation to examine endogenous CtBP2/ZFPM1 complex. The densitometric quantification is shown to the right of the blot (n=3 or 4 biologically independent animals for each group as shown in the blot, $p = 5.9 \times 10^{-5}$). **g,h.** Lactate/pyruvate ratio indicating cytosolic $NADH/NAD^+$ ratio in the liver of lean control or diet-induced (HFD, **g**, $p = 1.6 \times 10^{-3}$)/ genetically (ob, **h**) obese mice (n=5 for RD and HFD, n=4 for lean and n=3 for ob, biologically independent animals, 6-h fasted). **i.** The fatty acyl-CoA sensing capability of CtBP2/SREBP1 complex. Increasing concentrations of oleoyl-CoA (0, 50, 150, 500 μM) were added to the liver lysates from normal mice. **j.** CtBP2/SREBP1 complex formation in response to physiological stimuli.

Experimental settings are same as described in Fig. 3a-d and Supplementary Fig. 4a. Data are expressed as the mean ± SEM. * and ** denote p < 0.05 and p < 0.01 evaluated by unpaired two-tailed Student's t-test, respectively. Source Data are provided as a Source Data file.

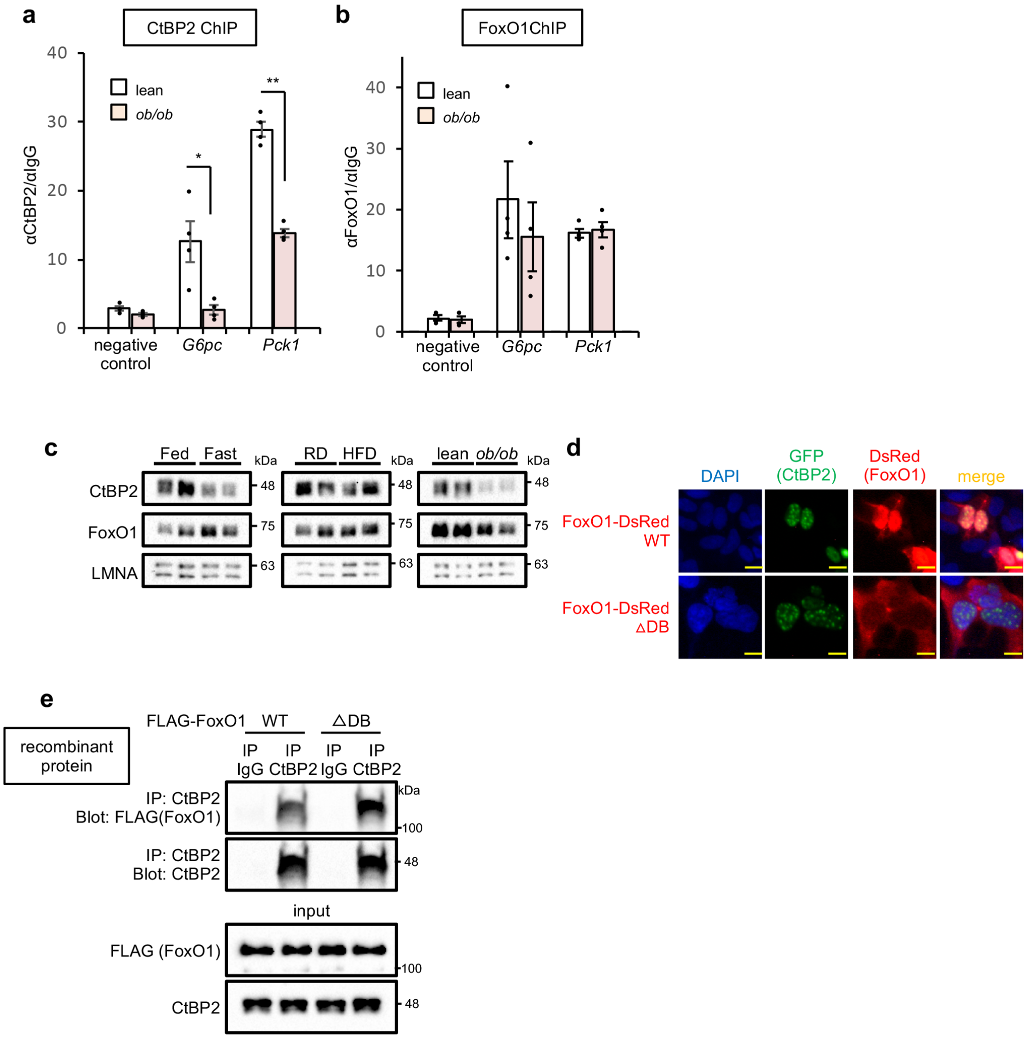

Supplementary Figure 5

Supplementary Fig. 5. Dissociation of CtBP2 from FoxO1 occurs in the nucleus.

a,**b.** CtBP2 (**a**, p = 0.018 and $1.9x10^{-5}$ for *G6pc* and *Pck1*, respectively) and FoxO1 (**b**) recruitment to promoter regions of gluconeogenic genes (n=4, biologically independent animals, 6-h fasted). ob: genetically obese mice. **c.** Liver nuclei were isolated from mice in different conditions (Fed: fed ad libitum, Fast: 24-h fast, RD: regular diet, HFD: diet-induced obesity, *ob/ob*: genetic obesity. Obese mice along with their control were fasted for 4-6 h). Expression levels of CtBP2 and FoxO1 in nuclei were determined. **d.** Either the wild-type FoxO1(WT)-DsRed or cytoplasmic FoxO1 mutant (△DB)-DsRed was expressed in HEK293 cells along with CtBP2-GFP. The yellow bars indicate 10 µm. **e.** The CtBP2/FoxO1 complex formation was analyzed using purified recombinant proteins in vitro. Either FLAG-wild-type (WT) FoxO1 protein or FLAG-FoxO1 mutant (△DB) protein was mixed with CtBP2 protein and immunoprecipitated with CtBP2 antibody. Data are expressed as the mean ± SEM. * and ** denote $p < 0.05$ and $p < 0.01$ evaluated by unpaired two-tailed Student's t-test, respectively. Source Data are provided as a Source Data file.

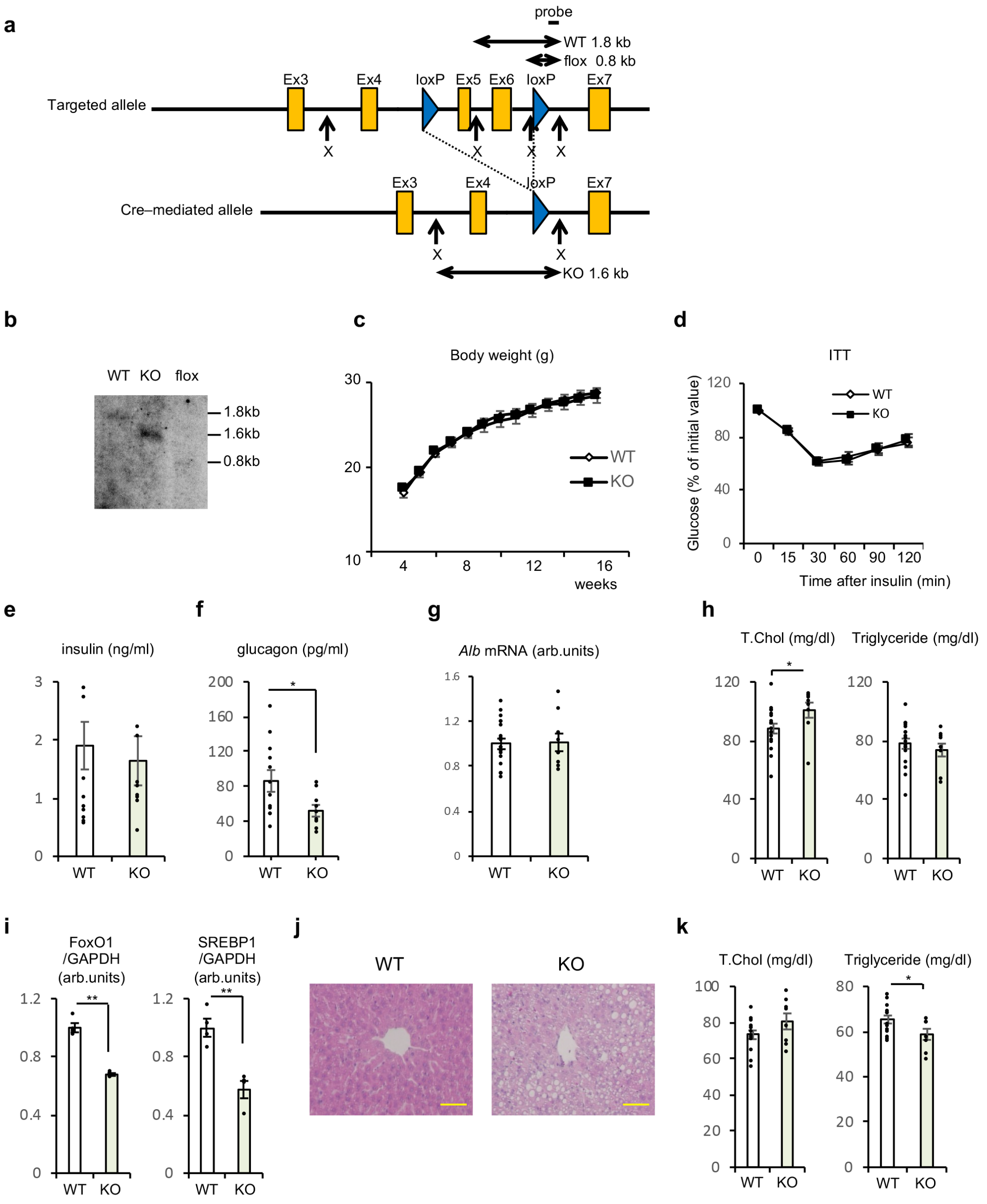

Supplementary Figure 6

Supplementary Fig.6 CtBP2 deficiency in liver leads to obesity-associated metabolic disturbances.

a. The targeting strategy for CtBP2 flox mice. X: XbaI. **b.** Southern blot analysis. **c.** Body weight (n=10, biologically independent animals). **d.** Insulin tolerance test (ITT) (n=12 for WT and n=6 for KO, biologically independent animals, 6-h fast). **e,f.** Plasma insulin (**e**) and glucagon (**f**, p = 0.041) levels following a 6-h fast (n=12 for WT and n=9 for KO, biologically independent animals). **g.** The expression levels of *Alb* gene in the liver tissues of either wild-type (WT) or liver-specific CtBP2-knockout (KO) mice (n=19 for WT, n=9 for KO, biologically independent animals, 6-h fast). **h**. Plasma lipid profile (n=18 for WT, n=9 for KO, biologically independent animals, 6-h fast, p = 0.046 for T.Chol). **i.** Expression levels of FoxO1 and SREBP1. Blots shown in Fig. 5g were quantified (n=4 biologically independent animals for each group, p = 6.9×10^{-5} and 2.6×10^{-3}, respectively). **j.** Representative Hematoxylin and Eosin stained sections of liver samples from mice on an MCD diet for a week. Yellow bars indicate 100 µm. **k**. Plasma lipid profile in mice on an MCD diet for a week (n=14 for WT and n=8 for KO, 6-h fast, p = 0.039 for Triglyceride). Data are expressed as the mean ± SEM. * and ** denote $p < 0.05$ and $p < 0.01$ evaluated by unpaired two-tailed Student's t-test, respectively. Source Data are provided as a Source Data file.

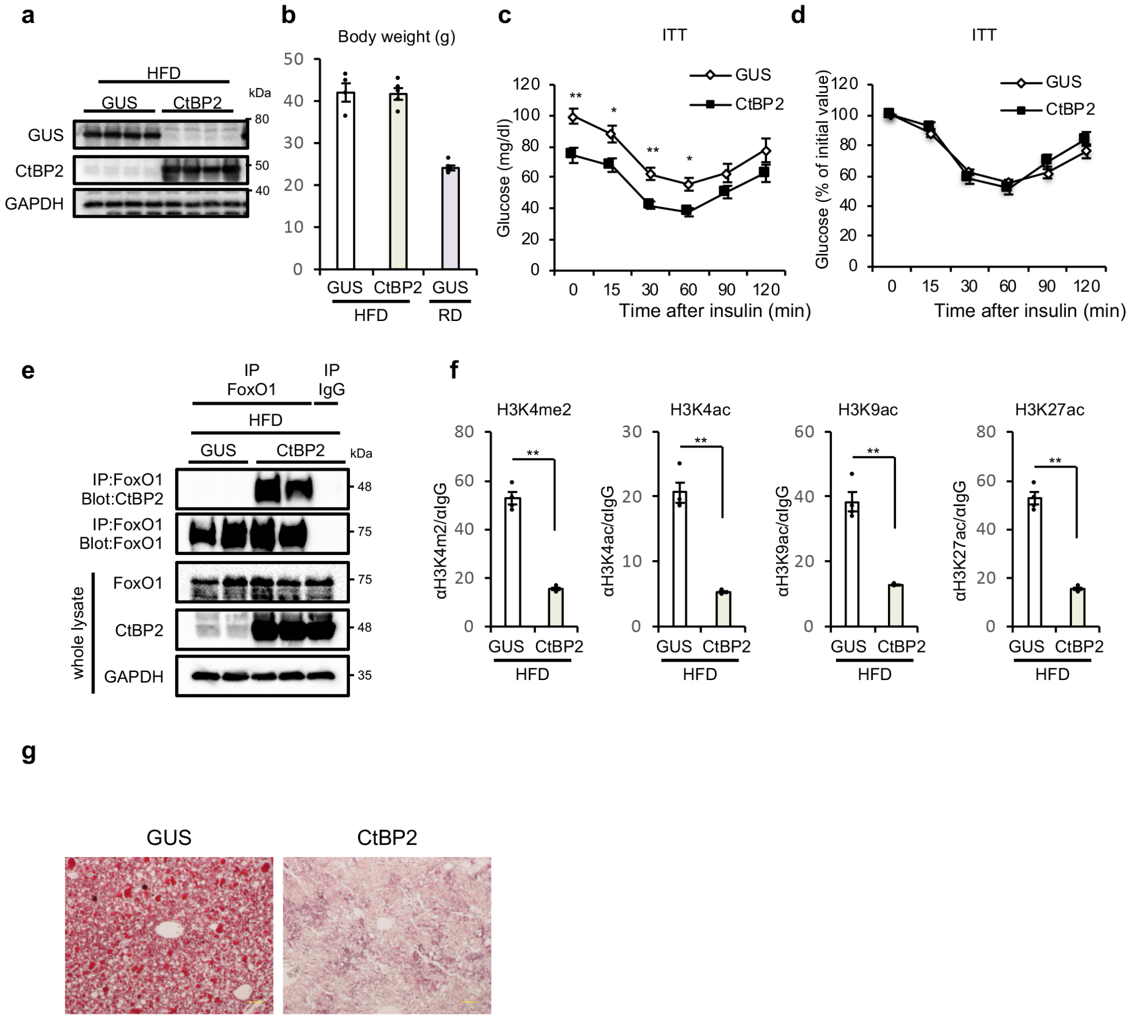

Supplementary Figure 7

Supplementary Fig. 7. CtBP2 gain of function in the liver of obese mice.

CtBP2 was exogenously expressed in the liver of diet-induced obese mice by adenoviral transduction (AdCtBP2), along with its control (AdGUS) (**a**,**b**,**f**: an overnight fast, **c**-**e**,**g**: 6-h fast). **a.** GUS and CtBP2 protein expression levels in liver. **b.** Body weights (n=4 for GUS/HFD, n=5 for CtBP2/HFD and GUS/lean, biologically independent animals). HFD: high fat diet, RD: regular diet. **c,d.**

Insulin tolerance test (ITT) (n=12 for GUS and n=10 for CtBP2, biologically independent animals, normalized as % of initial values in **d**, p = 1.5×10^{-3}, 0.013, 4.4×10^{-3}, 0.025 for 0 min, 15 min, 30 min, 60 min, respectively). **e.** CtBP2/FoxO1 complex induced by exogenous CtBP2 expression (biologically independent animals). **f**. Histone modifications at *G6pc* gene promoter (n=4 biologically independent animals for each group, p-values are as follows: 7.2×10^{-6} for H3K4me2, 6.5×10^{-5} for H3K4ac, 1.3×10^{-4} for H3K9ac, 7.3×10^{-6} for H3K27ac). **g**. Oil Red O staining of representative sections. Yellow bars indicate 100 µm. Data are expressed as the mean ± SEM. ** denotes $p < 0.01$ evaluated by unpaired two-tailed Student's t-test. Source Data are provided as a Source Data file.

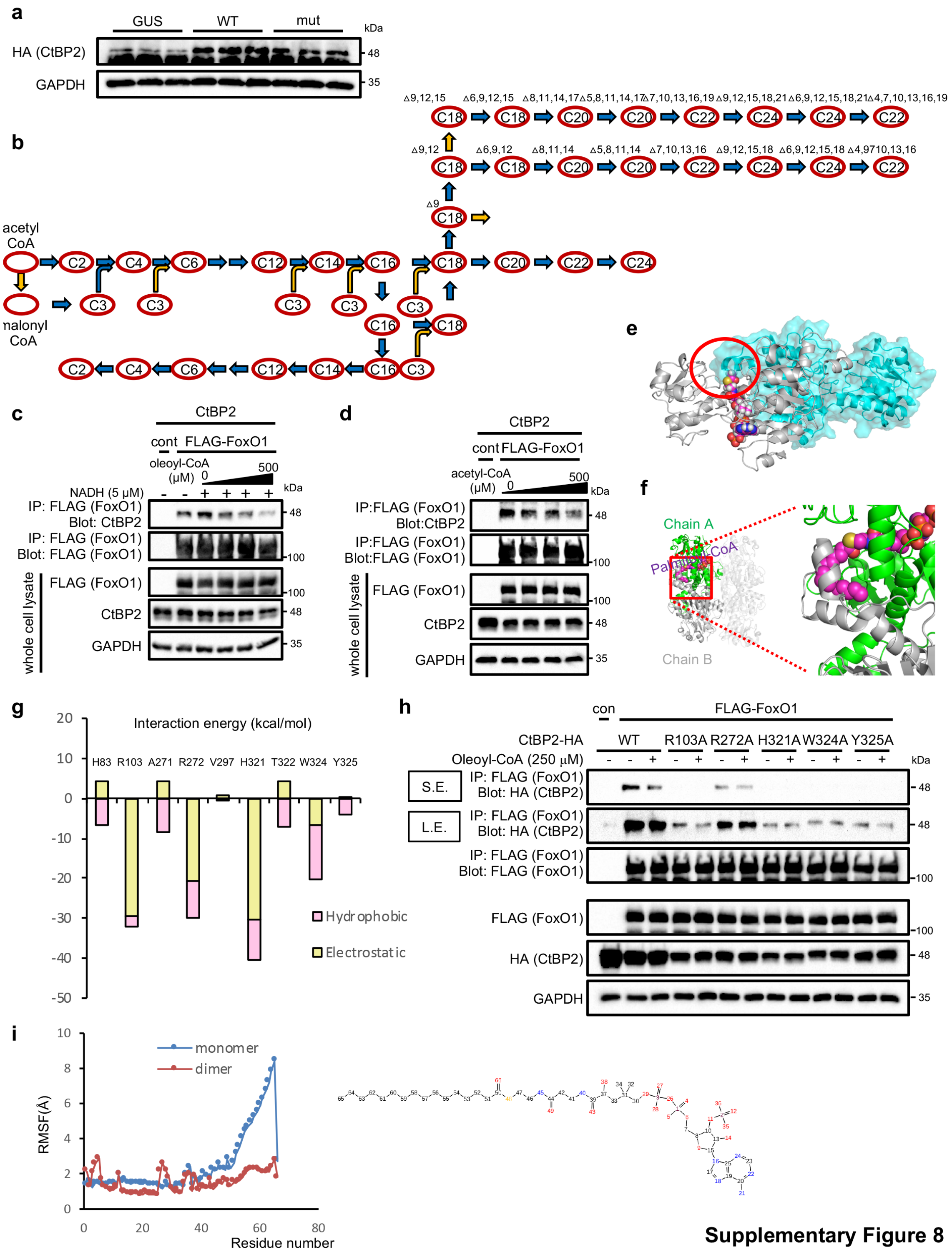

Supplementary Figure 8

Supplementary Fig. 8. The structure-function relationships in CtBP2 reveals critical coupling of metabolite-sensing with transcriptional regulation.

a. The protein expression levels of exogenously expressed wild-type (WT) CtBP2 and mutant lacking its Rossmann fold pocket (mut) in Fig. 7a were analyzed by detecting HA-tag fused to these two constructs (6-h fasted). **b.** Fatty acid synthesis in KEGG pathway analysis. Blue arrows indicate decreased activity by exogenous wild-type CtBP2 expression compared to GUS and Rossmann fold-mutant CtBP2. **c.** Cell lysates were obtained from HEK293 cells expressing CtBP2 with or without FLAG-FoxO1, and the CtBP2/FoxO1 complex was detected in the presence of the indicated concentrations of NADH (0 or 5 µM) and oleoyl-CoA (0, 50, 150, 500 µM). **d.** Increasing concentrations of acetyl-CoA (0, 50, 150, 500 µM) were added to the HEK293 lysates expressing CtBP2 and FLAG-FoxO1. **e.** Computer-assisted structural analysis of CtBP2 with acetyl-CoA. Red oval indicates the protruded acyl-chain of acetyl-CoA. **f.** Magnification of the interaction site between CtBP2 and palmitoyl-CoA obtained by our in silico structural modeling. **g**. Interaction energies estimated by the first-principles calculations FMO in Fig. 7j. **h**. Key residues in CtBP2 for the interaction with palmitoyl-CoA were mutated to alanine. Wild-type CtBP2-HA (WT) or the mutants along with a control plasmid or FLAG-FoxO1 were expressed in HEK293 cells and the cell lysates were immunoprecipitated with FLAG-tag in the presence or absence of oleoyl-CoA (250 µM). S.E.: shorter exposure, L.E.: longer exposure. **i**. The stability of palmitoyl-CoA was evaluated with monomeric form or dimeric form of CtBP2 by MD simulation. The

fluctuation of the palmitoyl-CoA residues in this simulation was quantified RMSF measurement. The residue numbering of the atoms is indicated to the right of the graph. High RMSF values indicate low binding affinities for CtBP2. Source Data are provided as a Source Data file.

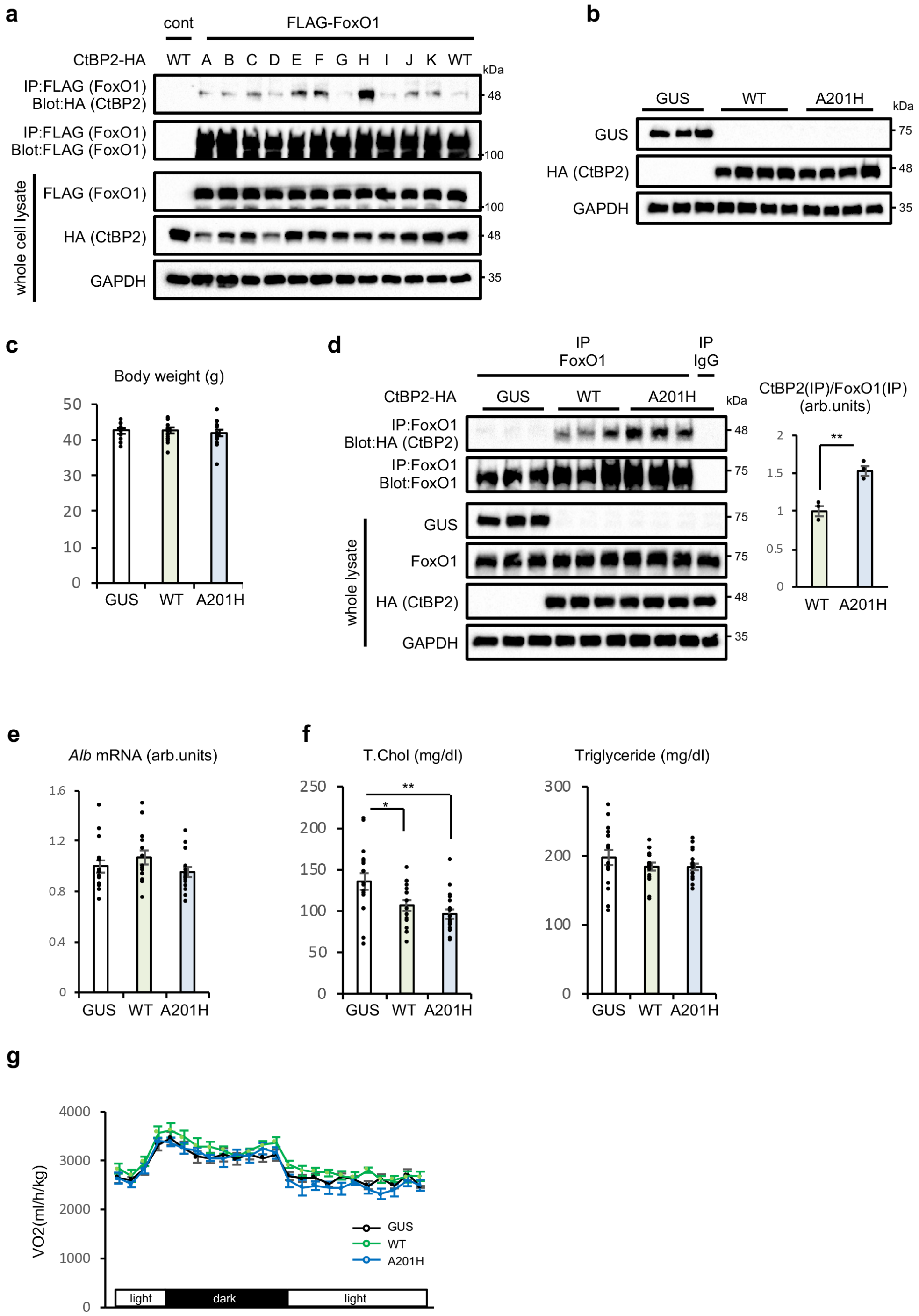

Supplementary Figure 9

Supplementary Fig.9. Dimerization is a key step for CtBP2 activation

a. A screen of mutations predicted to stabilize CtBP2 dimer formation. A series of CtBP2 mutants (A to K) as well as wild-type CtBP2 (WT) was expressed along with FLAG-FoxO1 in HEK293 cells and the interaction was analyzed. A: ASP136MET, B: ASP136TYR, C: ASP136HIS, D: ASN149ARG, E: GLN163TYR, F: SER164ARG, G: VAR165ARG, H: ALA201HIS, I: SER304MET, J: GLN327TYR, and K: SER330TRP. **b**-**g**: Adenovirus-mediated overexpression of wild-type CtBP2 (WT), ALA201HIS mutant CtBP2 (A201H) along with control (GUS) (3 days after transduction, b-f: overnight fasted)**. b**. The protein expression levels in Fig. 8d-g were analyzed by detecting HA-tag fused to these two constructs. **c.** Body weights (n=15 for GUS and A201H, n=16 for WT, biologically independent animals). **d.** Liver homogenates from mice (Fig. 8d-g) were subjected to co-immunoprecipitation to examine endogenous CtBP2/FoxO1 complex. The densitometric quantification is shown to the right of the blot (n=3 biologically independent animals for each group, $p = 4.7 \times 10^{-3}$). **e.** The expression of *Alb* gene (n=16 for GUS and WT, n=15 for A201H, biologically independent animals). **f**. Plasma lipid profile (n=17 for GUS and WT, n=18 for A201H, biologically independent animals, $p = 0.021$ and 1.6×10^{-3} for GUS vs WT and GUS vs A201H (T.Chol)). **g**. O_2 consumption (n=5, biologically independent animals). The dark and light phases are indicated by solid bars. Data are expressed as the mean ± SEM. * and ** denote $p < 0.05$ and $p < 0.01$ evaluated by unpaired two-tailed Student’s t-test, respectively. Source Data are provided as a Source Data file.

Supplementary Table 1. Primer list for our qPCR.

Gene		Sequence
Rplp0	Forward	5’-CACTGGTCTAGGACCCGAGAA-3’
	Reverse	5’-AGGGGGAGATGTTCAGCATGT-3’
Pck1	Forward	5’-CTGCATAACGGTCTGGACTTC-3’
	Reverse	5’-CAGCAACTGCCCGTACTCC-3’
G6pc	Forward	5’-CGACTCGCTATCTCCAAGTGA-3’
	Reverse	5’-GTTGAACCAGTCTCCGACCA-3’
Foxo1	Forward	5’-AACACACAGCTGGGTGTCAGG-3’
	Reverse	5’-GCATCTTTGGACTGCTCCTCAGT-3’
Ctbp2	Forward	5’-GCAGGACTTGCTATATCAGAGCGA-3’
	Reverse	5’-ATGCACCTTGCCTCATCTGCT-3’
Alb	Forward	5’-AGACGTGTGTTGCCGATGAGT-3’
	Reverse	5’-GTTTTCACGGAGGTTTGGAATG-3’
Gpam	Forward	5’-ACAGTTGGCACAATAGACGTTT-3’
	Reverse	5’-CCTTCCATTTCAGTGTTGCAGA-3’
Fasn	Forward	5’-AGAGATCCCGAGACGCTTCT-3’
	Reverse	5’-GCCTGGTAGGCATTCTGTAGT-3’
Scd1	Forward	5’-CCCGGGAGAATATCCTGGTTT-3’
	Reverse	5’-TCGATGAAGAACGTGGTGAAGT-3’
Ppargc1a	Forward	5’-GAAGTGGTGTAGCGACCAATC-3’
	Reverse	5’-AATGAGGGCAATCCGTCTTCA-3’
Srebf1c	Forward	5’-GGAGCCATGGATTGCACATT-3’
	Reverse	5’-GGCCCGGGAAGTCACTGT-3’
Mlxipl	Forward	5’-CACTCAGGGAATACACGCCTAC-3’
	Reverse	5’-ATCTTGGTCTTAGGGTCTTCAGG-3’
Pklr	Forward	TCAAGGCAGGGATGAACATTG
	Reverse	CACGGGTCTGTAGCTGAGTGG
Acly	Forward	ACCCTTTCACTGGGGATCACA
	Reverse	GACAGGGATCAGGATTTCCTTG
Crp	Forward	ATGGAGAAGCTACTCTGGTGCC
	Reverse	ACACACAGTAAAGGTGTTCAGTGGC
Cd36	Forward	AGATGACGTGGCAAAGAACAG
	Reverse	CCTTGGCTAGATAACGAACTCTG
Acadm	Forward	AGGGTTTAGTTTTGAGTTGACGG
	Reverse	CCCCGCTTTTGTCATATTCCG
