# Supplementary material for: The transcriptional corepressor CtBP2 serves as a metabolite sensor orchestrating hepatic glucose and lipid homeostasis
Source: Nat Commun. 2021 Nov 2;12:6315. doi: 10.1038/s41467-021-26638-5 (PMC8563733; doi:10.1038/s41467-021-26638-5)
Supplement: Supplementary file 3 — Description of Additional Supplementary Files [file 41467_2021_26638_MOESM3_ESM.docx]

**Description of Additional Supplementary Files**

**Title: Supplementary Data 1. Gene expression profile obtained from the RNA-seq analysis depicting the Rossmann fold-dependent transcriptional regulation by CtBP2.**

Description: Genes regulated by CtBP2 overexpression in the liver of obese mice in a Rossmann fold-dependent manner (Fig. 7b). Gene expression was analyzed by RNA-seq.

**Title: Supplementary Data 2. Residue scan to identify mutations to activate CtBP2.**

Description: Seventy-nine amino acid residues in the CtBP2 protein facing the dimerization interface were screened and the predicted changes of protein stability and dimerization affinity for all of the possible mutations were calculated (1660 mutations in total, Fig. 8a).

**Title: Supplementary Movie 1. MD simulation of the monomeric form of CtBP2 with palmitoyl-CoA.**

Description: The stick model and the green cartoon model show palmitoyl-CoA and the monomeric CtBP2, respectively. Palmitoyl-CoA was allowed to interact with monomeric form of CtBP2. The CoA moiety was fixed in the Rossmann fold while the acyl-chain moiety exhibited unrestricted movement.

**Title: Supplementary Movie 2. MD simulation of the dimeric form of CtBP2 with palmitoyl-CoA.**

Description: The stick model show palmitoyl-CoA, and the green and red cartoon models show the A and B chains of dimeric CtBP2, respectively. Palmitoyl-CoA was allowed to interact with dimeric form of CtBP2. The CoA moiety and the acyl-chain moiety were stabilized through interactions with the Rossmann fold in the CtBP2 A chain and the dimerization interface, respectively.
